# Re-Setting the Circadian Clock Using Exercise against Sarcopenia

**DOI:** 10.3390/ijms21093106

**Published:** 2020-04-28

**Authors:** Youngju Choi, Jinkyung Cho, Mi-Hyun No, Jun-Won Heo, Eun-Jeong Cho, Eunwook Chang, Dong-Ho Park, Ju-Hee Kang, Hyo-Bum Kwak

**Affiliations:** 1 Institute of Sports & Arts Convergence (ISAC), Inha University, Incheon 22212, Korea; choiyoungju0323@gmail.com (Y.C.); lovebuffalo@gmail.com (J.C.); gjwnsdnjs03@naver.com (J.-W.H.); cejeong97@naver.com (E.-J.C.); change@inha.ac.kr (E.C.); dparkosu@inha.ac.kr (D.-H.P.); johykang@inha.ac.kr (J.-H.K.); 2 Department of Kinesiology, Inha University, Incheon 22212, Korea; 77nodaji@hanmail.net; 3 Department of Pharmacology and Medicinal Toxicology Research Center, Inha University School of Medicine, Incheon 22212, Korea

**Keywords:** circadian rhythm, sarcopenia, exercise, mitochondrial function, skeletal muscle

## Abstract

Sarcopenia is defined as the involuntary loss of skeletal muscle mass and function with aging and is associated with several adverse health outcomes. Recently, the disruption of regular circadian rhythms, due to shift work or nocturnal lifestyle, is emerging as a novel deleterious factor for the development of sarcopenia. The underlying mechanisms responsible for circadian disruption-induced sarcopenia include molecular circadian clock and mitochondrial function associated with the regulation of circadian rhythms. Exercise is a potent modulator of skeletal muscle metabolism and is considered to be a crucial preventative and therapeutic intervention strategy for sarcopenia. Moreover, emerging evidence shows that exercise, acting as a zeitgeber (time cue) of the skeletal muscle clock, can be an efficacious tool for re-setting the clock in sarcopenia. In this review, we provide the evidence of the impact of circadian disruption on skeletal muscle loss resulting in sarcopenia. Furthermore, we highlight the importance of exercise timing (i.e., scheduled physical activity) as a novel therapeutic strategy to target circadian disruption in skeletal muscle.

## 1. Introduction

Skeletal muscle, which represents the largest organ of the human body, comprises approximately 40% of the total body mass and contains 50%‒75% of all body proteins. The main function of skeletal muscle is to maintain posture and produce movement that controls locomotion. Aside from this, skeletal muscle plays a central role in whole-body protein metabolism by serving as the main reservoir for amino acids in the absence of nutrient intake, allowing the maintenance of protein synthesis in other tissues of the body [1]. Overall, the functions of skeletal muscle are critical for systemic health, and reduced muscle mass and functions can lead to the development of many chronic diseases, including sarcopenia.

Sarcopenia, a progressive and generalized skeletal muscle disorder involving the loss of skeletal muscle mass and function [2] has long been associated with older adults [3,4] and can lead to adverse health outcomes, including physical disability, morbidity, and mortality [5,6,7,8]. However, in the past decade, circadian rhythm disruption, due to shift work or nocturnal lifestyle, has emerged as a prominent factor influencing the development of sarcopenia in addition to aging. In fact, a recent epidemiological study has suggested that shift workers who undertake variable rotating day and night shifts have an increased risk of sarcopenia [9]. Sleep deficiency (decreased sleep quality and/or sleep loss) or nocturnal lifestyle, which can contribute to circadian disruption, might also be involved in the development of sarcopenia [10,11]. Considering the significant increase in the number of shift workers worldwide [12,13,14] and individuals with a nocturnal lifestyle owing to an increasing 24-h culture, the association between circadian disruption and the prevalence of sarcopenia is an important area for future investigation.

Exercise is well-characterized as the major preventative and therapeutic strategy of sarcopenia that can attenuate and even reverse the loss of muscle mass and strength [15,16]. Previous studies have indicated that a balanced exercise program (at least 3 times/week), including resistance and endurance exercise training, could have positive effects on sarcopenia parameters and physical function. However, it remains unclear whether there is a specific timing of the day for exercise to trigger optimal training effects. Recently, emerging evidence has showed that exercise modulates the molecular circadian clocks in skeletal muscle [17,18] and exercise performance is variable throughout the day [19]. These findings showed a crosstalk between circadian rhythm and exercise, and it could be hypothesized that exercise timing (scheduled exercise) can assist in re-setting the clock and maximizing the beneficial effects of exercise associated with sarcopenia.

The aim of this review is to highlight the current literature regarding the potential mechanisms of exercise as a novel viable strategy, targeting circadian disruption associated with sarcopenia. Thus, we summarized the effect of circadian disruption on skeletal muscle disorder resulting in sarcopenia, along with the potential underlying mechanisms, while demonstrating when and why engaging in exercise might act as a therapeutic intervention to alleviate circadian disruption-induced sarcopenia.

## 2. Sarcopenia and Circadian Disruption

Increasing evidence indicates a possible association between circadian disruption and sarcopenia. Approximately 15‒30% of the global work population is engaged in shift work [12,13,14], and these workers have an increased risk of circadian disruption [20]. A prospective cohort study in South Korea showed a significant 1.7-fold increased risk in sarcopenia prevalence in shift workers than in those who had never experienced shift work [9]. In particular, they reported that irregularly scheduled shift work was more strongly associated with a higher prevalence of sarcopenia compared to regularly scheduled work. Indirect disruption of circadian rhythm due to sleep problems (duration, quality, and timing) or social jet lag can also have similar deleterious effects on skeletal muscle health in humans. Recent population- and laboratory-based studies have reported a U-shaped association between sleep duration and the prevalence of sarcopenia [21,22,23,24,25]. Furthermore, population- or hospital-based studies have found associations between components of sarcopenia and obstructive sleep apnea and decreased sleep quality [11,26]. More recently, a systematic meta-analysis concluded that sleep quality can predict the risk of developing sarcopenia [27]. Interestingly, later sleep timing, which can induce circadian misalignment as well as adversely affect sleep quality, has been shown to be associated with sarcopenia in middle-aged individuals [11]. Interaction between biological and societal clocks can lead to a chronic form of jetlag, depending on chronotype and social situation (i.e., social jet lag). Wittmann et al. have indicated that late chronotype is typically associated with a greater degree of misalignment between social and circadian time than other chronotypes [28]. Indeed, Yu et al. reported that the prevalence of sarcopenia is 6.7% higher in middle-aged Korean individuals with an evening chronotype rather than a morning chronotype [10]. Taken together, these findings indicated that the disruption of circadian rhythms is also related with an increased risk of developing sarcopenia in addition to aging. An overview of studies on the association between sarcopenia and circadian disruption is provided in Table 1.

## 3. Potential Mechanisms underlying Circadian Disruption Associated with Sarcopenia

### 3.1. Molecular Circadian Clock, Central and Peripheral Clocks, and Circadian Rhythm

The circadian system is governed by a central clock located in the suprachiasmatic nucleus (SCN) of the hypothalamus and peripheral clocks situated in other organs and tissues throughout the body, such as skeletal muscle. The central circadian clock is readjusted mainly by light inputs, the most important being time cue (i.e., Zeitgeber) [29]. Peripheral organs, such as skeletal muscle, have their own clocks, which are synchronized not only by the central clock [29] but also by an individual’s behaviors, including feeding or exercise [18,30]. Both central and peripheral clocks consist of a transcriptional-translational feedback loop (TTFL) known as the molecular clock. This feedback loop is mainly mediated by various activators, such as *Clock* (circadian locomotor output cycles kaput), *Bmal1* (brain and muscle Arnt-like protein-1), and their target genes, *Per1*, *Per2*, *Cry1*, and *Cry2*, which constitute negative repressor complexes that interact with *Clock* and *Bmal1* to inhibit *Per* and *Cry* gene transcription [31]. In addition to the main TTFL, *Rev-erbs* (nuclear receptor) and *RORs* (RAR-related orphan receptor) genes, which are the part of the core clock, link the second feedback loop by acting as the repressor and activator of *Bmal1* an *Clock* transcription, respectively [32,33]. As a result, the circadian rhythms produced by these molecular clocks oscillate over a period of approximately 24 h.

### 3.2. Mechanistic Pathways Connecting Circadian Disruption, Mitochondrial Dysfunction, and Sarcopenia

Although a direct relationship between circadian disruption and sarcopenia has not yet been elucidated in humans, several animal studies with mutations in clock genes have demonstrated that disruption of circadian rhythms can be detrimental to skeletal muscle health. Among clock genes, *Bmal1* and *Clock* are the key genes involved in the regulation of circadian rhythm. Global (whole-body) *Bmal1* knockout (KO) mice exhibited reduced muscle weight, body weight, and lifespan with disrupted circadian rhythms [34,35] and severe sarcopenia at 40 weeks of age [35]. Furthermore, core clock genes (*Bmal1* and *Clock*) mutant mice showed reductions in muscle force with mitochondrial dysfunction [36]. It should be noted, however, that these results are in contrast with those of the studies on a muscle-specific *Bmal1* KO model [37,38], showing no differences in muscle weight, muscle force, and life span between muscle-specific *Bmal1* KO and wild-type mice. Although it is also possible that muscle clock affects lipid metabolism to coincide with muscle protein turnover [39,40], skeletal muscle clock seems not to simply contribute to the progression of muscle atrophy [38]. Together, these results suggest that disruption or weakening of the circadian rhythm via deletion of the central clock gene, not by the muscle-specific clock gene, might play an important role in the pathogenesis of sarcopenia.

Recent evidence has also suggested that mitochondria might play a critical role in circadian disruption-related sarcopenia. In skeletal muscles, mitochondria are an important and essential organelle involved in metabolic regulation and production of adenosine triphosphate (ATP) for muscle contractibility and plasticity. Many studies have revealed that mitochondria play a key role in the pathogenesis of sarcopenia [41,42,43], and mitochondrial dysfunction, along with aging, has been extensively studied as a contributor to sarcopenia [44,45]. However, regulation of mitochondrial function via the circadian system has also gained interest as emerging evidence indicates that circadian disruption is associated with mitochondrial dysfunction [46,47]. The importance of circadian rhythms for mitochondrial processes is made evident by the genetic disruption of the molecular clock. For example, circadian mutant mice (*Bmal1* and *Clock*) displayed reduced muscle mitochondrial volume, respiratory function, and peroxisome proliferator-activated receptor-gamma coactivator-1 alpha (PGC-1α) level with subsequent reduction in contractile muscle force [36]. Similarly, *Clock* mutant mice (*Clock* △19) exhibited decreased mitochondrial function including decrease in levels of PGC-1α, mitochondrial transcription factor-A (Tfam) protein, and mitochondria content with reduced exercise capacity [48]. In particular, PGC-1α is often referred to as the master regulator of mitochondrial biogenesis, and thus, these findings suggest that reduced PGC-1α in circadian mutant mice might contribute to decrease in mitochondrial content and respiration [36,48]. Moreover, Liu et al. found that mice with depletion of PGC-1α in skeletal muscle showed reduced clock gene expression and disrupted circadian rhythm [49]. They found that PGC-1α can stimulate the expression of clock genes, such as *Bmal1* and *Rev-erb*α via coactivation of the ROR family, which is part of the core clock of the second feedback loop. In turn, these studies suggest that PGC-1α likely plays a pivotal role in the circadian TTFL, involved ultimately in mitochondrial functioning. Together, these findings indicate that decreases in the protein content and expressions of clock genes and PGC-1α might help in elucidating the effects of circadian disruption on mitochondrial dysfunction, leading to sarcopenia.

In addition to circadian clock-mediated regulation, mitochondrial function in skeletal muscle exhibits an intrinsic circadian rhythm [46,47]. A recent study in human skeletal muscle biopsy samples demonstrated that *Bmal1* exhibited significant variation over time with peak expression at around midnight (23:00 h), whereas *Per2* exhibited a trough expression at midnight, and that mitochondrial function (e.g., mitochondrial oxidative capacity) also displays circadian rhythm and peaks in the late afternoon [46]. Interestingly, the same study found time-dependent variations in proteins involved in mitochondrial dynamics; the levels of mitochondrial fission 1 (Fis1; fission mediator) displayed significant time-dependent differences similar to those of rhythm in mitochondrial oxidative capacity, whereas levels of PTEN-induced kinase 1 (PINK-1; a marker of mitophagy) showed an opposite rhythm as compared to Fis1. However, no rhythm was observed in the mitochondrial content (e.g., mitochondrial DNA) and mitochondrial biogenesis (e.g., PGC-1α) in human skeletal muscle. Taken together, these data indicate that skeletal muscle mitochondria are influenced either by the circadian clock or the intrinsic circadian rhythms, which suggests association between the circadian system and mitochondrial metabolism. Disturbances in the rhythm of the mitochondrial function of skeletal muscle could play a critical role in the pathology of skeletal muscle.

## 4. Novel Aspects of Exercise as a Zeitgeber for Re-setting the Clock against Sarcopenia

### 4.1. Exercise, Molecular Circadian Clock Gene, and Circadian Synchronization

As previously stated, skeletal muscles have their own circadian rhythm and can be entrained by physical activity for skeletal muscle [18,29] and light for the SCN. With respect to association between exercise and muscle clock genes, several studies have demonstrated that exercise, such as wheel running or forced treadmill running, in nocturnal rodents alters the expression of core clock genes in skeletal muscle [50] and could entrain circadian rhythms [51,52,53]. In addition, scheduled exercise training resulted in a significant shift in clock gene expression in the skeletal muscle of mice [18], supporting that exercise might be an external time cue (zeitgeber) for the clock in skeletal muscle.

Zambon et al. also examined the effect of a single bout of exercise on gene regulation in human muscle biopsy samples [17]. Their results showed that three core circadian clock genes, *Bmal1*, *Cry1*, and *Per2*, were upregulated by resistance exercise in one leg compared with the non-exercise leg. Human studies also have established that exercise has significant circadian phase-shifting effects (Table 2) [54,55,56,57,58,59,60,61,62] and can easily entrain an individual to a shifted light-dark and sleep/wake schedule. In particular, several studies showed that morning exercise accelerated phase advances, while evening exercise accelerated phase delays in the circadian rhythm [54,56,57,59], highlighting the importance of exercise timing in differentiating between phase-delaying and phase-advancing effects. With respect to the phase-shifting effect of exercise, especially in shift work, Eastman et al. examined whether timed exercise during consecutive night shifts could phase delay the circadian rhythm of humans to align with a daytime sleep schedule [55]. In this experiment, the participants cycled for 15 min every hour during the first three out of eight consecutive night shifts. As a result, exercise produced large phase delays in core temperature rhythm regardless of their chronotypes (morningness or eveningness), suggesting that exercise can be a powerful determinant of phase shift. Moreover, Youngstedt et al. revealed that early morning exercise with moderate intensity (65‒75% heart rate reserve at 4:10‒5:40 am) following late evening bright light (22:10‒23:40 pm) can have an additive circadian phase-shifting effect [61]. Interestingly, recent work by Thomas et al. indicated that individuals with late chronotypes, experiencing severe circadian misalignment, may benefit from phase advances with 5 days of morning or evening exercise, whereas evening exercise may exacerbate circadian misalignment in early chronotypes [62]. Together, these findings suggest that exercise, and more importantly, the timing of exercise could act as a circadian time cue by changing the phase of circadian clock, and thereby, personalized exercise timing prescription could alleviate circadian disruption.

### 4.2. Impacts of the Scheduled Exercise (Exercise Timing) on Mitochondrial Function and Muscle Performance

In addition to the circadian phase-shifting effects of exercise, timing of exercise (i.e., circadian time of exercise) can contribute to the time-point of peak physical performance. Previous research has revealed that there are diurnal variations in skeletal muscular performance [19,63] and oxidative capacity [46,47]. Indeed, studies have consistently reported that skeletal muscle torque, strength, and power in humans increased in the late afternoon, between 16:00 and 18:00 pm, compared with in morning [19,63]. In addition, mitochondrial oxidative capacity in human skeletal muscle has been shown to exhibit circadian changes, with a peak in the late evening (23:00 pm) [46,47]. Previous studies have reported that the peak aerobic performance is also seen later in the day [64,65,66], which might be partly responsible for the diurnal fluctuations in response to mitochondrial function.

Evidence from our group and other studies has shown that both acute and chronic exercises lead to increased mitochondrial function of skeletal muscle in both animals and humans [67,68]. A recent study by our group revealed that a single bout of exercise improved mitochondrial function, including mitochondrial O_2_ respiration and Ca^2+^ retention capacity, in skeletal muscles of rats [68]. However, it has not been completely resolved whether any specific time of exercise with a concomitant peak in mitochondrial function increases the effect of exercise in terms of muscle function. It is clear that the time of exercise training (i.e., morning or evening exercise) in human has different outcomes on muscle mass and muscle performance [69,70,71,72]. A recent study from our group showed that evening exercise (19:00 h) in the cardiac muscles of rats, and not morning exercise (07:00 h), increases PGC-1α mRNA levels, suggesting that mitochondrial biogenesis could be influenced by circadian time of exercise [73]. However, there is still very little information regarding the effect of circadian exercise on skeletal muscle mitochondria. Further studies will be needed to investigate whether a specific time of exercise could maximize both skeletal muscle mitochondrial function and overall exercise performance.

## 5. Conclusions

Disruption of circadian rhythm is one of the critical factors that leads to sarcopenia in various individuals, especially in people with shift work, social jetlag, and sleep disorder [9,10,11,21]. Although a direct relationship between circadian disruption and sarcopenia has not been identified in humans, several studies in different mouse models of clock gene deficiency have demonstrated that circadian disruption associated with mitochondrial dysfunction might contribute to the pathology of skeletal muscle [36]. Indeed, mitochondria play a central role in the progression of sarcopenia [42,43], and emerging evidence has revealed that mitochondrial function in skeletal muscle has intrinsic circadian rhythms [46,47], possibly involved in the molecular mechanisms of sarcopenia. Taken together, there is ample evidence to highlight the important role of circadian rhythm in the skeletal muscle system, suggesting restoring the circadian rhythm as a possible therapeutic intervention in prevalent sarcopenia.

**Figure 1:**
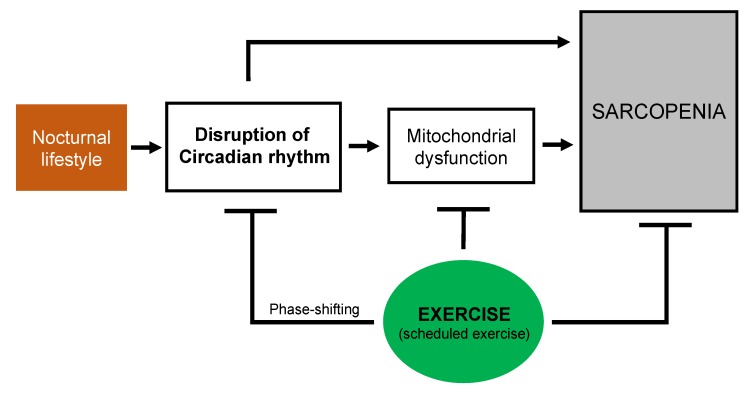
Schematic overview of the potential beneficial effects of exercise on circadian disruption and mitochondrial dysfunction associated with sarcopenia.

Exercise has been suggested to be one of the most effective strategies against the loss of muscle function and muscle mass [15]. Consideration of the circadian phase-shifting effects of exercise might help in rendering the circadian exercise intervention more effective in re-setting the clock and optimizing the beneficial effects of exercise associated with sarcopenia. In particular, personalized exercise timing could be prescribed as an effective tool to prevent and treat sarcopenia in various individuals who work shifts or have a nocturnal lifestyle. As a consequence, exercise, and more importantly scheduled exercise, might be useful in preventing and treating circadian disruption-related sarcopenia (Figure 1). Further research should elucidate whether a specific time of exercise would improve skeletal muscle function and the mechanisms through which scheduled exercise could optimize skeletal muscle function, particularly the mechanisms involving mitochondrial function.

## Figures and Tables

**Table 1:** Sarcopenia and circadian disruption in humans.

Design	Participant	Main Findings	Reference
Laboratory-based study	48 postmenopausal women (61 ± 6 y)	Sleep duration was correlated with muscle strength (*r* = −0.43, *p* = 0.002) and skeletal muscle mass (*r* = −0.39, *p* = 0.007). In addition, long sleepers (>9 h) had significantly lower values for skeletal muscle mass (*p* = 0.03) and muscle strength (*p* = 0.01).	[21]
Population-based study	488 older adults (76.8 ± 6.9 y)	Compared with the normal sleep duration (6‒8 h), short sleepers (<6 h) had a nearly 3-fold increased risk of sarcopenia (OR: 2.76; 95% CI: 1.28–5.96), while long sleepers (>8 h) had a nearly 2-fold increased risk of sarcopenia (OR: 1.89; 95% CI: 1.01–3.54).	[22]
Population-based study	1620 adults (40‒69 y)	Evening type, when compared with morning type, was significantly associated with sarcopenia (OR: 3.16; 95% CI: 1.36–7.33) after adjusting for confounding factors.	[10]
Population-based study	1196 adults (68 ± 4 y)	The adjusted OR for low muscle mass was 2.8 for men with poor sleep quality (95% CI: 1.1–6.7) and 4.3 for men with poor sleep efficiency (95% CI: 1.2–15.1). In woman, poor sleep quality was found to be associated with reduced grip strength and low appendicular lean mass.	[23]
Population-based study	607 older adults (60‒90 y)	Compared with the normal sleep duration (6‒8 h), short sleepers (<6 h) had an over 4-fold increased risk of sarcopenia (adjusted OR: 4.24; 95% CI: 1.75–10.30), whereas long sleepers (>8 h) had an over 3-fold increased risk of sarcopenia (adjusted OR: 3.50; 95% CI: 1.39–8.80).	[25]
Population-based study	16148 adults (44.1 ± 0.2 y)	Compared with the 7 h of sleep duration, the OR (95% CI) for sarcopenia of the long sleepers (≥9 h) was 1.589 (1.100–2.295) after adjusting for confounding factors.	[24]
Population-based study	915 middle-aged adults (45‒65 y)	PSQI score was associated with sarcopenia (OR: 1.10; 95% CI: 1.02–1.19); ORs of sleep latency and later mid-sleep time with sarcopenia were 1.14 (0.99–1.31) and 1.54 (0.91–2.61), respectively.	[11]
Hospital-based study	334 patients with sleep problem (61.9 ± 10.4 y)	AHI, an index of the severity of sleep apnea, was correlated positively with the skeletal muscle mass index and negatively with skeletal muscle density in both men and women.	[26]
Population-based study	9105 workers	Compared with the group that had never experienced shift work, the OR (95% CI) for sarcopenia of the shift work group was 1.7 (1.5–1.9); the association remained even after adjusting for confounding factors.	[9]

OR, odds ratios; CI, confidence interval; PSQI, Pittsburgh Sleep Quality Index; AHI, apnea-hypopnea index.

**Table 2:** Circadian phase-shifting effects of exercise in humans.

Participant	Protocol	Main Findings	Reference
17 young men (20‒30 y)	(1) Control session without exercise (2) Night exercise with moderate intensity of 3-h duration (40‒60% VO_2peak_)	A single nocturnal exercise phase delays circadian rhythms of melatonin and thyrotropin secretion.	[54]
8 young men(20‒30 y)	(1) Control session without exercise (2) Night exercise with a 3-h bout of moderate intensity (40‒60% VO_2peak_) or a 1-h bout of high intensity (75% VO_2peak_)	Nocturnal high-intensity exercise as well as moderate-intensity exercise phase delays circadian rhythms of melatonin and thyrotropin secretion.	[56]
16 adults (19‒41 y)	(1) Control group without exercise (2) Exercise group: 15 min every hour (50‒60% HRmax) during the first three of eight consecutive night shifts (with daytime sleep)	Exercise during night shifts facilitates circadian rhythm of core temperature phase delays.	[55]
46 young adults (20‒28 y)	(1) Control session without exercise (2) Exercise sessions: morning, afternoon, or night exercise with moderate intensity of 2-h duration (HR of 140 beats/min)	Afternoon and night exercise elicits phase delays of the circadian rhythm of plasma melatonin.	[57]
8 young (20‒32 y) and 10 older (55‒73 y) adults	(1) Control session without exercise (2) Night exercise with low intensity of 3-h duration (40‒60% VO_2peak_)	Nocturnal exercise accelerates phase delays, whereas early evening exercise accelerates phase advances in older and young adults.	[58]
38 young men (20‒30 y)	(1) Control group without exercise (2) Exercise groups: morning, afternoon, evening, or night exercise with high intensity of 1-h duration (75% VO_2peak_)	Morning exercise elicits phase delays and evening exercise elicits phase advances.	[59]
22 young men (22.0 ± 1.8 y)	(1) Control group without exercise (2) Exercise groups: morning or evening exercise of 2-h duration (interval exercise at 65‒75% HRmax)	Both morning and evening exercise elicits a similar extent of phase-delay shifts. But the falling phase shifted by 1.0 h only after evening exercise.	[60]
6 young adults (18‒30 y)	(1) Late night bright light alone (2) Late night treadmill exercise alone (interval exercise at 65‒75%HRR) (3) Late night bright light followed by early morning exercise	Late night bright light followed by early morning exercise has additive phase-delaying effect on the circadian rhythm of melatonin compared with exercise alone.	[61]
52 young adults (18‒45 y)	Five days of morning or evening exercise with high intensity for 30 min (75% VO_2peak_)	Morning (compared with evening) exercise advances the phase of the circadian rhythm. In particular, in people with a late chronotype, both morning and evening exercise induces phase advances.	[62]

VO_2peak_, peak oxygen uptake; HR, heart rate; HRR, heart rate reserve.
